# EEG Correlates of the Flow State: A Combination of Increased Frontal Theta and Moderate Frontocentral Alpha Rhythm in the Mental Arithmetic Task

**DOI:** 10.3389/fpsyg.2018.00300

**Published:** 2018-03-09

**Authors:** Kenji Katahira, Yoichi Yamazaki, Chiaki Yamaoka, Hiroaki Ozaki, Sayaka Nakagawa, Noriko Nagata

**Affiliations:** ^1^School of Science and Technology, Kwansei Gakuin University, Sanda, Japan; ^2^Research Center for Kansei Value Creation, Kwansei Gakuin University, Sanda, Japan

**Keywords:** Flow experience, Objective measurement, EEG, mental arithmetic task, FM theta

## Abstract

Flow experience is a subjective state experienced during holistic involvement in a certain activity, which has been reported to function as a factor promoting motivation, skill development, and better performance in the activity. To verify the positive effects of flow and develop a method to utilize it, the establishment of a reliable measurement of the flow state is essential. The present study utilized an electroencephalogram (EEG) during an experimentally evoked flow state and examined the possibility of objective measurement of immediate flow. A total of 16 participants (10 males, 6 females) participated in the experiment that employed a mental arithmetic task developed in a previous study. Post-trial self-report of the flow state and EEG during task execution were measured and compared among three conditions (Boredom, Flow, and Overload) that had different levels of task difficulty. Furthermore, the correlations between subjective flow items and EEG activity were examined. As expected, the ratings on the subjective evaluation items representing the flow state were the highest in the Flow condition. Regarding the EEG data, theta activities in the frontal areas were higher in the Flow and the Overload conditions than in the Boredom condition, and alpha activity in the frontal areas and the right central area gradually increased depending on the task difficulty. These EEG activities correlated with self-reported flow experience, especially items related to the concentration on the task and task difficulty. From the results, the flow state was characterized by increased theta activities in the frontal areas and moderate alpha activities in the frontal and central areas. The former may be related to a high level of cognitive control and immersion in task, and the latter suggests that the load on the working memory was not excessive. The findings of this study suggest the possibility of distinguishing the flow state from other states using multiple EEG activities and indicate the need for other physiological indicators corresponding to the other aspects of flow experience.

## Introduction

In any task, for better performance, performing at one's full potential in individual situations and obtaining the required skills in advance are important. In general, the development of an individual's ability through training or performance, specific to each activity, enables better performance. On the other hand, an optimal psychological state that contributes to these goals can be a common promotional factor across various types of activity. The most promising optimal psychological state is the flow experience proposed by Csikszentmihalyi (2000). According to Csikszentmihalyi (2000), who originally proposed this concept, flow is the “holistic sensation that people feel when they act with total involvement.” The state of flow is characterized by a sense of intrinsic reward experienced during immersive engagement in an activity. Flow was investigated in a broad spectrum of activities; for example, chess playing (Abuhamdeh and Csikszentmihalyi, 2009) and rock climbing (Fave et al., 2003). The common characteristics of flow (Nakamura and Csikszentmihalyi, 2009) are summarized as two conditions of flow and six subjective states of flow as shown in Table 1.

**Table 1:** The components of flow experience.

**The conditions of flow**
Balance between challenge and skill
Clear goals and feedback
**The subjective states of flow**
Intense concentration
Merging of action and awareness
Loss of reflective self-consciousness
A sense of control
Distortion of temporal experience
Experience of intrinsic reward

Flow was originally found in studies that examined the phenomenological aspects of autotelic or intrinsically motivated activities (Nakamura and Csikszentmihalyi, 2009). Therefore, flow is regarded as the optimal experience that promotes intense commitment or an individual's development in a certain activity. Previous research has revealed that flow seems to enhance several aspects of activities; for example, motivation (Csikszentmihalyi et al., 1993; Jackson et al., 1998), development (Carli et al., 1988; Nakamura, 1988), and performance (Jackson et al., 2001; MacDonald et al., 2006).

By definition, flow is considered to trigger a feeling of intrinsic reward. Therefore, it is assumed that those who experience the state of flow in a specific activity experience an increase in their motivation to engage in that activity. Prolonged influences of flow on motivation for specific activities were found in a study conducted by Csikszentmihalyi et al. (1993), which longitudinally investigated the activities of teenagers who were talented in specific areas. Students who experienced more flow in their talented areas tended to be involved in activities in these areas 4 years later, which suggested the relationship between flow and continuous commitment to activities. In the field of sports, a study on elderly athletes revealed a positive relationship between the state of flow and intrinsic motivation (Jackson et al., 1998).

Experiencing flow makes activities intrinsically rewarding, which enables development of skills related to an activity by encouraging increased commitment. Moreover, the balance between skills and challenges required for experiencing flow provides an optimal level of challenge that develops existing skills (Vygotsky, 1978). Therefore, flow is considered to foster the development of skills in the long term. Several studies have shown the relationship between flow and achievement in the high school context (Carli et al., 1988; Nakamura, 1988).

Furthermore, flow is associated with the quality of performance of specific tasks. Jackson et al. (2001) reported that the state of flow during competitive sports correlated with quality of performance measured both subjectively and objectively. MacDonald et al. (2006) revealed that flow experienced by students when working on a group composition task was related to specialists' assessment of the quality of produced musical compositions. This may be due to reduced self-consciousness when experiencing flow, which may decrease performance anxiety (Fullagar et al., 2013) and encourage performers to focus on their task.

Given the positive influences of flow described above, it is expected that increasing the experience of flow promotes individuals' development and enhances motivation and quality of performance in each specific situation. Recently, research has been conducted aiming to utilize these outcomes of flow. In the field of web design, it is assumed that experiential flow determines user behavior in the context of the computer-mediated environment. For example, the relationship between flow and online consumer behavior was investigated in a previous study (Smith and Sivakumar, 2004). The attributes of the website influencing the experience of flow were also examined (Huang, 2003). In the context of computer game research, enjoyment of video games is considered to be explainable in terms of the flow experience (Weber et al., 2009). Researchers have not only employed game playing as an appropriate task to investigate flow (Keller and Bless, 2008), but also explored the factors of game playing that impact the evocation of flow (Johnson and Wiles, 2003). In the context of music education, the flow experience is drawing attention as a factor that promotes musical learning. In addition to the development of applications to enhance the flow experience of adults (Nijs et al., 2012) and children (Addessi et al., 2015) in musical activities, individual resources that contribute to evoking flow including social factors have been investigated (Bakker, 2005). Furthermore, in the field of human-computer interaction, it has been pointed out that flow is a promising indicator for dealing with the state of mind of users who actively engage in certain activities (Leman et al., 2010).

Although the utility of flow has been found in various fields, further research is necessary to establish how beneficial outcomes can be obtained by evoking the flow experience. For such efforts, various types of measures would be required to assess whether the developed methods increase flow. Several psychological measures have been developed to evaluate the flow experience subjectively. However, the self-report approach requires interrupting an activity and taking time to collect responses, which makes it difficult to measure an immediate state of flow during ongoing activities. To make this possible, it would be effective to establish a method to measure the state of flow based on an objective index. With respect to examples of indicators based on observation of behavior, Custodero (2005) proposed indices to measure the flow of infants and school-age children based on observable nonverbal behavior, while Addessi et al. (2015) developed an observation-based coding system to measure the occurrence of flow.

Several previous studies have attempted to measure the flow state objectively using physiological and neurological measures. Klasen et al. (2011) conducted an fMRI study to assess brain activity during the first person's game task. They analyzed the contents of the game play to identify events that were likely to evoke the state of flow and revealed the activities of multiple brain regions during those events. However, the influence of the movements generated for game play was suggested for some of those activities. Ulrich et al. (2014) examined brain activity when flow was experienced using fMRI in a mental arithmetic task, which minimized the influence of bodily movements. The brain activities were compared in three conditions, which were different in terms of task difficulty: Boredom, Flow, and Overload conditions. The relationship between brain activity and self-reported flow experience was also examined. They concluded that increased activity in the inferior frontal gyrus and putamen reflected a deeper sense of cognitive control and coding of reward-related information, and decreased activity in the medial prefrontal cortex and amygdala represented a loss of self-referential processing and negative arousal.

Research using fMRI described above has clarified neural activity as consistent with the theory of flow; however, given our purpose of assessing the method used to evoke the state of flow, low temporal resolution of imaging techniques is problematic. To measure the immediate flow state and its time course during the experimental task, other indicators with a higher temporal resolution are considered to be more effective. de Manzano et al. (2010) measured heart rate, respiration, and electromyogram from professional pianists during a piano performance, and identified physiological activities related to self-reported flow experience. Among the physiological measures, electroencephalogram (EEG) has been reported to be related to various brain functions; therefore, EEG is considered suitable for exploring indices related to flow, which is a combination of a variety of subjective states. However, EEG activity when experiencing flow state has not been fully investigated.

The purpose of this study was to clarify EEG activities corresponding to an experimentally evoked flow state. Using the experimental paradigm of the previous study, which adopted the mental arithmetic task (Ulrich et al., 2014), several subjective states—including flow—were experimentally produced, and EEG data were compared between these states. Furthermore, the relationship between the self-reported flow experience measured by the psychological scale and the EEG data was examined. These investigations were expected to reveal clues about the pattern of EEG activity characterizing the flow state, which contributes to developing a real-time objective measurement method of flow state. There are no comparable data because no study has yet measured EEG data when flow was evoked using this experimental paradigm. Therefore, in this research, no specific hypotheses were formulated, and exploratory investigation was conducted across multiple electrode positions and EEG frequency ranges.

## Methods

### Participants

A total of 16 right-handed university students (10 males, 6 females; average age: 21.9 ± 1.1 years) participated in this study, and were paid 1,000 yen per hour. The duration of the experiment varied from individual to individual (3–4.5 h). The study was conducted in accordance with the local ethics committee of Kwansei Gakuin University. Under normal circumstances, the local ethics committee does not request a full review and any approval procedure if researchers are following the committee guidelines. Since this study adhered to the guidelines of the committee, no specific approval procedure was carried out. The instructions for the experimental procedure were provided to the participants both orally and in writing, and written informed consent was obtained prior to the experiment.

### Experimental design

The participants performed mental arithmetic tasks in several task conditions that differed in terms of calculation difficulty. The task and experimental manipulation in the present experiment almost replicated the approach used in a previous study by Ulrich et al. (2014).

**Figure 1:**
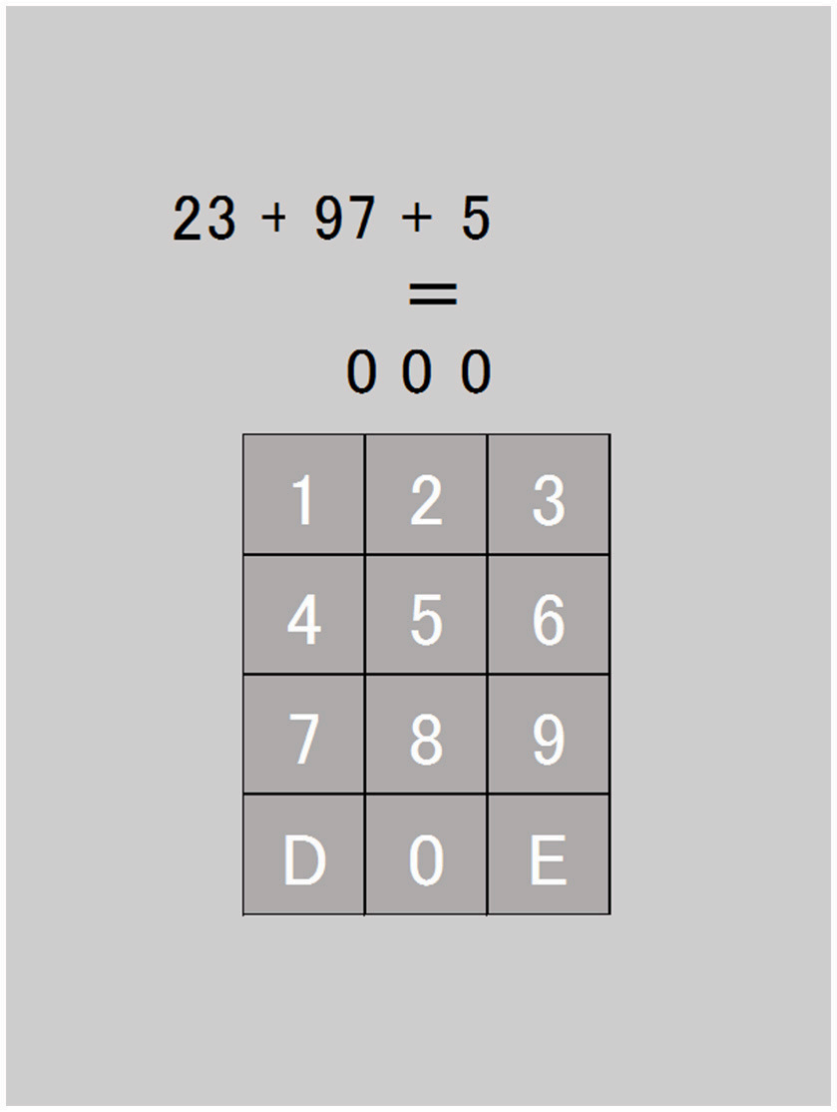
On-screen keyboard used in the experiment.

In the employed arithmetic ask, participants mentally summed the numbers and provided the result, which was designed to always have three digits. To minimize body movement, the participants entered the result on the keyboard displayed on the screen (Figure 1) using a trackball. The trial was started by presenting the mathematical expression on the screen; the participants then entered the result digit-by-digit and submitted it by pressing the “E” button. There was no feedback regarding the accuracy of the calculation in order not to cause differences in motivation due to the ratio of positive to negative feedback.

Three conditions with different task difficulty were used: Boredom, Flow, and Overload. The Boredom condition was characterized by a low-difficulty task and provided the less challenging situation to participants. The Flow condition employed dynamic adjustment so that the task difficulty was always adjusted to participants' level of skill, thus providing a situation with an appropriate level of challenge. The Overload condition also employed a similar adjustment, while an over-high-difficulty task was generated. All participants performed the arithmetic task in all three conditions. The task difficulty in each condition was determined or adjusted as follows.

The Boredom condition always included two numbers to be summed. The first number was randomly selected from 100 to 109, and the second number was from 1 to 9. To make the task difficulty constantly low, the summation of these two numbers was always between 101 and 110.

In the Flow condition, the task difficulty was automatically and continuously adjusted in each trial to fit participants' skills. Each trial started with a task difficulty that suited participants' levels of skill identified in advance. The adjustment of task difficulty was made based on the results of the last two tasks. When the last two results were correct, the task difficulty increased in two ways as described below. If the last summand of the latest mathematical expression had one digit, the last summand in the next calculation changed to a two-digit number so that task difficulty increased. In the case of the last summand with two digits, the next calculation was added by an additional one-digit summand. For every single adjustment, either of these two changes was applied. Thus, these two types of adjustment occurred alternatively. On the other hand, when two successive results were wrong, the task difficulty decreased in the reverse manner of the above-mentioned method.

In the Overload condition, the initial task difficulty was three levels higher than that in the Flow condition. The task difficulty increased when at least three out of the last five trials had correct results, and decreased if at least four out of the last five results were incorrect. The task difficulty did not fall below the initial level.

The experiment included three task blocks for each task condition as well as three Rest blocks. The trial was repeated for each task block that lasted 184 s. The Rest blocks were introduced to measure the resting state where no mathematical problem was presented and the participants maintained a gaze fixation. To eliminate the effect of block order, two types of block sequence were employed, and these sequences were counterbalanced across participants.

Prior to the experiment, the participants performed two preliminary tasks to familiarize them with using a trackball and to measure their level of skill for the arithmetic task. In the former task, the participants performed the arithmetic task with the task difficulty used in Boredom condition for 5 min. In the latter task, the participants performed the arithmetic task used in Flow condition for 5 min, and the level of calculation of the individual participants were identified as an average task difficulty derived from the last 25% calculations.

Since it is desirable to maintain constant time intervals between the onset of arithmetic tasks when analyzing EEG data, a trial protocol was changed from that used in the previous study. More specifically, the time interval between entering the last result and the presentation of the current mathematical expression was varied to keep the duration of single trials constant. The preliminary experiment with five participants investigated the influence of this modification of trial protocol and confirmed that it had no effect on the subjective ratings. Each task block always included eight trials due to this modification. The first trial in each block was regarded as a preparatory step for the participants to enter the flow state and was excluded from the analyses.

### Measurement

As the performance data of the arithmetic task, the calculation results submitted in each trial were recorded. Furthermore, the time duration from the presentation of the mathematical expression to the entering of the results was measured to examine the time required by the participants for the calculation.

After each task block, the participants were asked to complete the questionnaire shown in Table 2 that measured flow state in that block. This questionnaire consisted of nine items used by Ulrich et al. (2014) and two items of perceived challenge and goal that were suggested to be the components of the experience of flow. Ten items were rated on a seven-point Likert-scale ranging from 1 (“I do not agree at all”) to 7 (“I completely agree”). Participants responded to one item that assessed their subjective sense of time on a visual analog scale ranging from 0 (“very short”) to 100 (“very long”).

**Table 2:** Items for measuring subjective flow experience.

Item 1	I would love to solve math calculations of that kind again
Item 2	I was strongly involved in the task
Item 3	I was thrilled
Item 4	The task was boring
Item 5	I had the necessary skill to solve the calculations successfully
Item 6	Task demands were well matched to my ability
Item 7	During the task, I was concerned with other task-irrelevant issues
Item 8	During the task, my consciousness was completely focused on solving the math calculations
Item 9	The task was challenging
Item 10	I was heading toward the goal
Item 11	Subjective experience of time

The EEG data were recorded throughout the task using the BioSemi Active-Two system (BioSemi Inc., The Netherlands). A whole-scalp, 64-channel active electrode EEG array electrodes according to the International 10/20 EEG system and extra 6-channel set on the face collected EEG data at a sampling rate of 1024 Hz.

### Analysis

As for performance data, the percentage of correct answers and response time were averaged for each task condition for each participant. The effect of the task condition on these two types of performance data was examined using analysis of variance.

Regarding subjective experience, rating scores on each item of the questionnaire measuring the state of flow were averaged across the three blocks of each task condition. Differences between each pair of task conditions were examined for each item using analysis of variance. Furthermore, to investigate the aspects of flow measured by the employed questionnaire, a factor analysis was performed on the rating data.

EEG data were analyzed using EEGLAB (Version 13.4.4b) and FieldTrip (Build 20140522) working on MATLAB (Version R2013b, the MathWorks, Inc., Massachusetts, USA). The EEG data were down-sampled to 256 Hz, filtered with 1 and 100 Hz bandpass. An independent component analysis was performed on the EEG data to identify independent components corresponding to noises that were to be manually rejected using visual inspection. Additionally, the Laplacian filter was applied. Each trial was extracted from the preprocessed data, and trials including eye blinks and noisy channels where potentials exceeded ±100 μV were discarded. The averaged rejection rate was 2.2% (*SD* = 4.2) for all the participants.

A time-frequency analysis was applied to the data from each task block. The analysis was performed using Morlet wavelet analysis available on the FieldTrip toolbox. A total of 7 cycles determining the width of wavelets was used. In this analysis, the wavelet's center frequencies range from 1 to 100 Hz in steps of 1 Hz, and the time window was moved from 10 s before to 25 s after the trial onset in steps of 5 ms. The analyzed data were averaged for each task condition for each participant.

Based on the averaged data, four frequency ranges were extracted (delta: 1–3 Hz, theta: 4–7 Hz, alpha: 10–13 Hz, beta: 14–30 Hz). Separate ROI analyses were conducted focusing seven areas (Frontocentral: AFz, Fz, FCz; Left frontal: AF3, F3, F7, FC3; Right frontal: AF4, F4, F8, FC4; Left central: C3, C5, CP3, CP5; Right central: C4, C6, CP4, CP6; Left occipital: P3, P5, PO3, PO7; Right occipital: P4, P6, PO4, PO8) for each frequency range. The data of each trial included the EEG data after answering the mathematical problem. To focus on EEG activity while solving the computational problem, amplitudes during the interval from 1 s after the trial onset to average response time in each condition (Boredom condition: 5.5 s, Flow condition: 14.8 s, Overload condition: 17.2 s after the trial onset) were averaged for each area for each frequency range. The amplitudes in each task condition were assessed in terms of the amount of changes based on the data from the Rest blocks. To test the effect of task condition on amplitudes, analysis of variance was performed.

## Results

### Behavioral data

The performance of mental arithmetic task was assessed in terms of the percentage of correct answers. The average percentage of correct answer was 99.7% in the Boredom condition, 54.4% in the Flow condition, and 16.9% in the Overload condition. A significant effect of task condition was seen [*F*_(2, 30)_ = 476.2, *p* < 0.001], and the results of the multiple comparisons using Bonferroni method ensured significant differences between all the conditions (*p* < 0.001 for all pairs). The averages of the response time were 5.5 s in the Boredom condition, 14.8 s in the Flow condition, and 17.2 s in the Overload condition. Significant differences between the conditions were also revealed for the response time [*F*_(2, 30)_ = 613.7, *p* < 0.001], and pair-wise differences were significant (*p* < 0.001 for all pairs).

**Figure 2:**
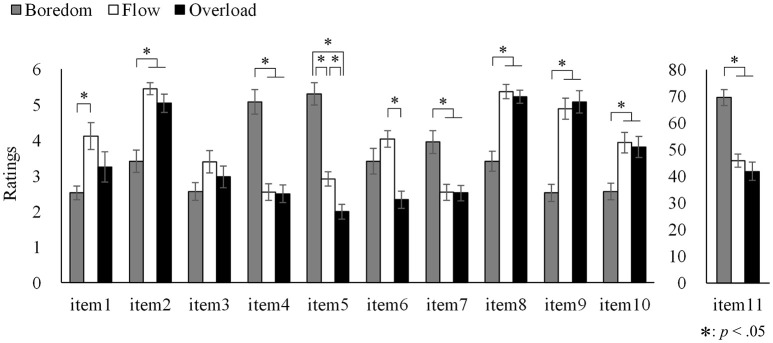
Comparison of the ratings of subjective flow items in the Boredom, Flow. and Overload conditions. Error bars indicate standard error. ^*^p < 0.05.

The influence of task conditions on participants' subjective rating was investigated. Individuals' rating data were averaged across the three blocks of each task condition with respect to each item. Analyses of variance including task condition as a factor were conducted separately on 11 items. The results are shown in Figure 2. The significant effects of task condition were found for all the items [minimum *F*_(2, 30)_ = 4.076, *p* < 0.05]. Multiple comparisons using Bonferroni method revealed similar trends for items 2, 8, 9, and 10. That is, the ratings for these items in the Boredom condition were significantly lower than those in the other two conditions. Items 4, 5, 7, and 11 showed an opposite trend, in which the ratings were significantly higher in the Boredom condition than in the other two conditions. Additionally, for item 5, the rating in the Overload condition was significantly lower than that in the Flow condition. For items 1, 3, and 6, the ratings were the highest in the Flow condition; however, significant differences between task conditions were observed only for item 1 (Boredom vs. Flow) and item 6 (Flow vs. Overload).

A factor analysis using the maximum-likelihood method and promax rotation was performed on 10 items excluding item 11 that asked about the subjective sense of time. The analysis extracted two factors based on the Kaiser-Guttman criterion, which explained 70.0% of the total variance. The first factor includes the items concerning task demand (items 4, 5, 9) and the items describing immersion into the task (items 2, 7, and 8). In particular, item 5 (“I had the necessary skill to solve the calculations successfully”) showed a strong relation solely to this factor, suggesting that it can be interpreted as a factor of the level of challenge. The second factor includes the antecedent condition of flow (item 6), the positive experience during task execution (items 3 and 10), and the consequent motivation (item 1). In particular, since item 6 (“Task demands were well matched to my ability”) had a moderate association with this factor alone, it seemed adequate to interpret this factor as a factor of balance between challenge and skill (Table 3).

**Table 3:** Result of the factor analysis of subjective flow items.

	**Factor**	
	**Level of challenge**	**Balance between challenge and skill**
Item 8	0.970	0.525
Item 7	−0.899	−0.502
Item 9	0.868	0.667
Item 2	0.853	0.588
Item 4	−0.797	−0.517
Item 5	−0.671	−0.114
Item 3	0.472	0.883
Item 1	0.533	0.815
Item 10	0.670	0.795
Item 6	0.088	0.448

### EEG data

**Figure 3:**
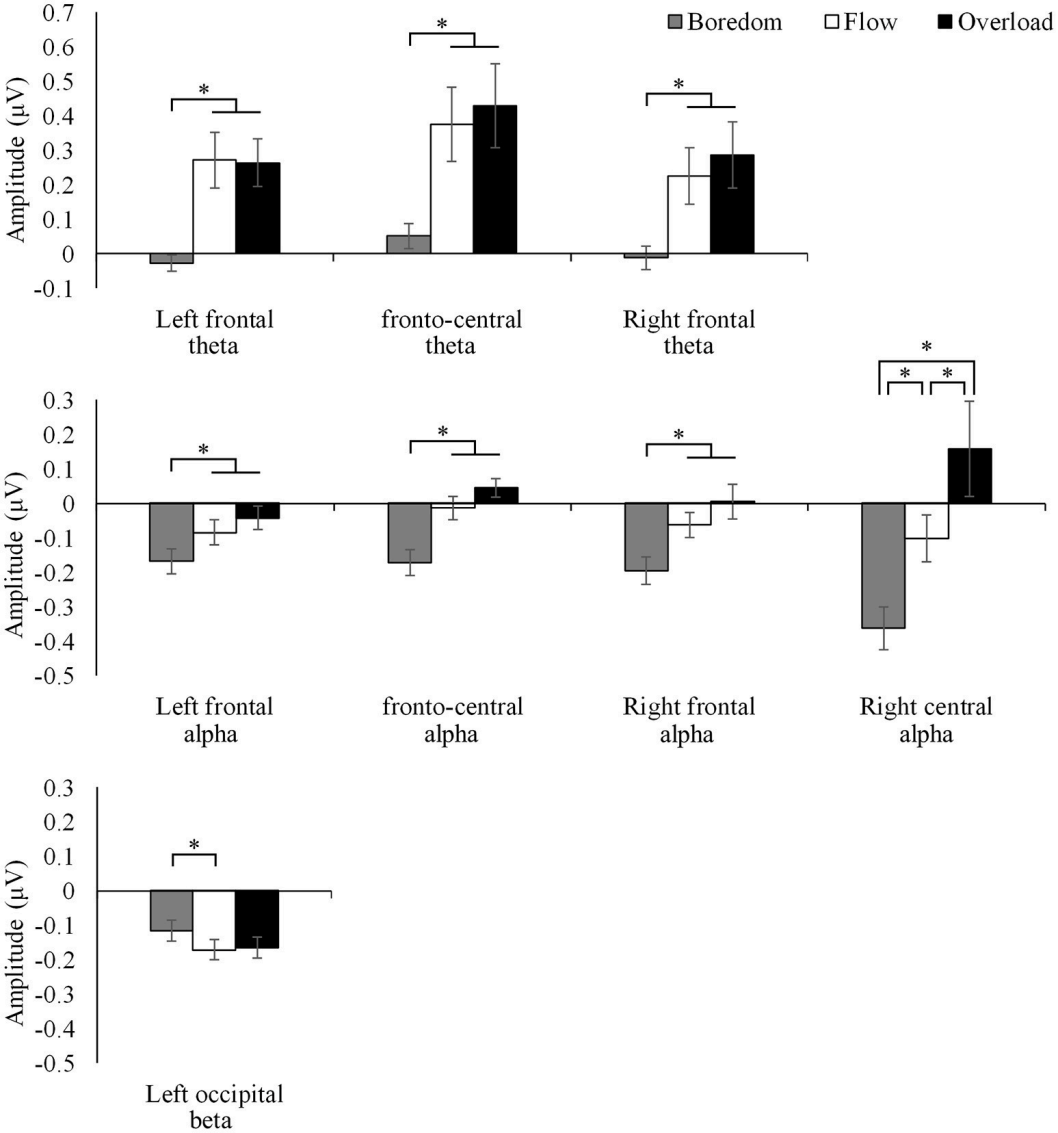
Comparison of the amplitude of EEG activity between the Boredom, Flow, and Overload conditions. Error bars indicate standard error. ^*^p < 0.05.

As a result of examining the effect of the task condition on the four EEG rhythms in the seven areas, all the EEG rhythms showed a significant effect of the task condition in one or more areas [minimum *F*_(2, 30)_ = 4.017, *p* < 0.05]. The results of the multiple comparisons using Bonferroni method revealed several significant differences between the conditions, which are shown in Figure 3. In both theta and alpha activities, significant differences were observed between boredom and all other conditions in the frontal areas (*p* < 0.05 for all pairs), and the amplitude of the Boredom condition was smaller than the other two conditions. In addition, alpha activity at the right central area showed significant differences in all pairs of conditions (*p* < 0.05 for all pairs), the Boredom condition showed the smallest amplitude and the Overload condition showed the largest amplitude. Furthermore, for the left occipital beta activity, the amplitude of the Boredom condition was significantly larger than that of the Flow condition (*p* < 0.05).

### Correlations between subjective flow and EEG data

Focusing on the EEG rhythms with significant differences between the conditions—theta and alpha in the frontal area, alpha in the right center, and beta in the left occipital—correlations between the amplitude of the EEG activities and subjective rating data of flow were examined (Table 4). When calculating correlation coefficients, data were pooled over conditions and individuals. The amplitudes of the theta and alpha activities in the frontal area showed a similar pattern of significant correlation (including correlation with trend toward significance) as a whole (except for the alpha activity at the left frontal). In particular, items 2, 8, 9, and a factor of the level of challenge showed positive correlations with many EEG rhythms. On the other hand, items 4, 5, 7, and 11 showed negative correlations with many rhythms. Item 10 showed a positive correlation only with the theta activities in frontal areas and item 1 and a factor of balance between challenge and skill showed a positive correlation only with the theta activity in the left frontal area. Items 3 and 6 had no correlation with any of the EEG rhythms. The left occipital beta activity tended to be different from other rhythms and showed a negative correlation only with item 9.

**Table 4:** Correlations between EEG rhythms and subjective flow items (bolded = *p* < 0.05, bolded italic = *p* < 0.10, corrected with false discovery rate method).

	**Theta**			**Alpha**				**Beta**
	**Fronto-central**	**Left frontal**	**Right frontal**	**Fronto-central**	**Left frontal**	**Right frontal**	**Right central**	**Left occipital**
Item 1	0.281	***0.339***	0.263	0.112	−0.098	0.018	0.142	−0.221
Item 2	0.251	**0.436**	0.249	***0.347***	0.222	**0.463**	***0.339***	−0.169
Item 3	0.101	0.210	0.085	0.108	0.048	0.089	0.221	−0.089
Item 4	–***0.375***	–**0.430**	–***0.325***	–***0.309***	−0.086	−0.273	−0.242	0.278
Item 5	–***0.320***	–**0.409**	–***0.329***	–**0.495**	−0.248	–**0.520**	–**0.377**	0.192
Item 6	0.173	0.117	0.162	−0.023	−0.119	−0.088	−0.143	−0.040
Item 7	−0.207	–***0.321***	−0.182	–**0.386**	−0.267	–**0.389**	–***0.295***	0.092
Item 8	***0.304***	**0.391**	0.278	**0.377**	0.171	***0.364***	0.210	−0.233
Item 9	**0.402**	**0.475**	***0.347***	***0.365***	0.117	***0.339***	0.285	–***0.320***
Item 10	**0.422**	***0.353***	***0.346***	0.269	0.023	0.155	0.184	−0.229
Item 11	–***0.325***	–***0.327***	−0.253	–***0.314***	−0.126	–***0.324***	–***0.306***	0.214
Level of challenge	***0.344***	**0.435**	***0.304***	**0.407**	0.192	**0.402**	0.278	−0.238
Balance between challenge and skill	0.269	***0.325***	0.230	0.156	−0.005	0.094	0.192	−0.189

## Discussion

### Behavioral data

In this research, the experimental paradigm of Ulrich et al. (2014) was used to experimentally evoke flow and other conditions. The results of the mental calculation tasks were 99.7% for the Boredom condition, 54.4% for the Flow condition, and 16.9% for the Overload condition. Compared to previous research, the rate of correct answers in the Overload condition was lower; however, those of the other two conditions were at similar levels (98.4, 63.2, 31.0%, respectively, in Ulrich et al., 2014). In terms of subjective evaluation data, many items showed similar trends to previous research; for example, the reverse U-shape tendency in items 1, 3, and 6 was also observed in the results of Ulrich et al. (2014). Based on these facts, the experimental paradigm in this study created a similar task situation as the previous research, and the subjective state experienced in each condition can also be regarded to resemble those in the previous research.

To investigate which aspects of flow were measured by the scale used in this research, factor analysis was performed on subjective evaluation data. The results showed a two-factor structure, and these factors can be interpreted as the level of challenge and the balance between challenge and skill. Each factor was a combination of several flow components. The factor of the level of challenge included aspects related to the perception of task difficulty level and concentration on the task and is considered to represent a high level of challenge that induces holistic participation in the task. The factor of balance between challenge and skill included different aspects, such as appropriate task difficulty as an antecedent condition of flow, positive subjective state during flow, and motivation for activities in the future, and is considered the factor that expresses the state of flow comprehensively. Items 1, 3, and 6 that were used to calculate a composite variable as the representatives of flow in the previous research were included in the factor of balance between challenge and skill; this also confirmed the agreement between this study and the previous one regarding the measurement of subjective states.

### EEG activity corresponding to the flow state

To clarify the EEG activity that is specific to the flow state, the differences between the conditions of the amplitude of the EEG activities were examined using analysis of variance. As a result, significant differences between the conditions were observed in the theta and alpha activities at the frontal area, the alpha activity at the right central area, and the left occipital beta activity. Among them, the left occipital beta activity might be an artifact caused by the effects of bodily movement, which could not be completely eliminated; therefore, only the theta and alpha activities are discussed below.

The frontal theta activity showed significantly smaller amplitude in the Boredom condition compared to the other conditions, and showed the same level of activity in the Flow and Overload conditions. The frontal theta activities are called the FM theta, and have been shown to be related to cognitive control (Cavanagh and Frank, 2014) and concentration (Lagopoulos et al., 2009). Since flow occurs in a situation in which the maximum ability of an individual is required, it is considered that maximum cognitive control is manifested in the state of flow. In addition, immersion in a task is an important component of flow. Therefore, it is considered that the high theta activity in the Flow condition reflected these subjective states accompanying the flow experience. However, a high level of cognitive control and deep commitment to the task are also applicable to the Overload condition; this may be the reason there was no difference between the Flow and Overload conditions.

On the other hand, the alpha activity at the frontal and right central areas showed a tendency to increase gradually based on the degree of task difficulty. Especially, at the right central area, there were significant differences in the alpha activity not only among the Boredom condition and the other two conditions, but also between the Flow and Overload conditions. It has been pointed out that the increase in alpha activity in the parieto-occipital area is related to the load on working memory (Tuladhar et al., 2007), and it is possible that the observed alpha activity in this study may reflect the difference in the load on working memory that arose from the different task difficulty levels. Unlike the result of the theta activity, which suggested that the similar level of cognitive control to the Overload condition occurred in the Flow condition, the alpha activity seemed to reflect the task difficulty *per se* that brought about a high level of cognitive control, which made it possible to distinguish the Flow and Overload conditions.

In summary, first, the differences in EEG activity between the Boredom condition and other conditions were clearly demonstrated. Specifically, the Boredom condition was characterized by a lower frontal theta activity, suggesting less cognitive control, and a lower frontal and right central alpha activity suggesting a lower load on the working memory. Second, the results suggested that the alpha activity may clarify the difference between the Flow and Overload conditions. The Flow condition was characterized by lower alpha activity compared to the Overload condition. Putting together the data on the alpha and theta activity, as the EEG indicators that are specific to the flow state, the combination of a high theta activity suggesting a high level of cognitive control and immersion in a task with a moderate alpha activity suggesting a relatively low load on working memory may be effective.

### Correlation between EEG and subjective rating data

As a result of the correlation analysis of the subjective evaluation of flow and EEG data, all EEG rhythms showed a similar correlation with the subjective rating data, except for the left occipital beta activity. The factor of the level of challenge was correlated with both the theta rhythm and the alpha rhythm, which can be interpreted in terms of the factor being representative of evaluation of task difficulty (“The task was boring,” “I had the necessary skill to solve the calculations successfully,” and “The task was challenging”) and immersion state (“I was strongly involved in the task,” “During the task, I was concerned with other task-irrelevant issues,” and “During the task, my consciousness was completely focused on solving the math calculations”). It is thought that the degree of task difficulty indirectly brought about cognitive control and immersion in the task indicated by the theta activity, and directly affected the load on the working memory indicated by the alpha activity. Therefore, these tendencies are consistent with the interpretation obtained from the comparison of the differences in conditions.

On the other hand, only theta activity correlated with the factor of balance between challenge and skill that included item 1, “I would love to solve math calculations of that kind again,” and item 10, “I was heading toward the goal.” It has been reported that the power of the frontal theta activity increases when the working memory is manipulated successfully (Itthipuripat et al., 2013), and it is considered that this EEG rhythm is related to not only cognitive control itself, but also its smooth performance. Given that the factor of balance between challenge and skill and its representative items (1 and 10) are related to the positive experiences in the appropriate condition of the level of challenge, it would be reasonable that this factor showed a positive correlation with the theta activity.

### Objective measurement of flow state using EEG activity

Compared to the study by Ulrich et al. (2014) using fMRI, the present study seems to provide complementary findings. They found indices of brain activity correlated with a single psychological measure that is at its peak under the Flow condition. On the other hand, the present study found several indices of EEG activity correlating with different aspects of the subjective rating of the flow, and may collectively discriminate all of the conditions of the Boredom, Flow, and Overload. With respect to the association with brain functions, Ulrich et al. (2014) connected their indices with neural activities that reflect positive experience in the flow state, that is, deeper sense of cognitive control and decreased negative emotions. The EEG indices found in this study do not seem to reflect emotional outcome in the flow state, but rather the state of cognitive activity (the level of cognitive effort and cognitive load) during task execution. Taking all of the above into consideration, Ulrich et al. (2014) and our research may provide neurological indicators corresponding to different aspects of the flow experience.

Since this study used only a mental arithmetic task, it is unclear whether the identified EEG activity will work as an index of flow in other types of activities. However, the findings of this research provide plausible clues about the features of internal processing while flow is experienced in a given task, that is, the state in which holistic involvement in the task can be achieved without adverse effects of stress. Specific EEG activities corresponding to flow among various tasks may differ from each other; however, the features of internal processing reflected by those EEG activities may share the properties mentioned above. On the other hand, it should be noted that the results of this study do not fully explain the contents of flow that will be experienced in the fields of more complex activities. According to Biasutti (2017), musicians in the flow state challenge new attempts and their experiences often involve discovery. It is unlikely that these aspects of the flow state were experienced in the task used in this research, therefore, it is expected that EEG studies targeting more creative activities will be conducted in the future.

Objective measurements of the flow state using EEG activity can be useful, especially when behavior is not expressed at an observable level. However, the necessity for an observational approach remains because it is expected that the activities that enable EEG measurement without disturbing the occurrence of flow are limited, especially when the participants are children. Future studies employing both observational methods and EEG measurements may yield fruitful results as an attempt to clarify the relationship between the behavioral aspects and the state of internal processing of flow.

### Problems and implications for future research

When interpreting the results of this research, it is possible to point out the differences in the trial time course among the task conditions. In the experimental paradigm of this study, several different phases can be identified in the trial, which are (a) from the time the calculation problem is presented until a participant completes the mental calculation, (b) from the time a participant starts the input of the mentally calculated answer until the “E” button is pressed and the answer is submitted, and (c) from the time the answer to a problem is submitted until the next computational problem is presented. Such a task structure is inevitable in the experimental paradigm using this mental arithmetic task; however, this may have influenced the difference in physiological responses among the conditions. For example, the time until a participant completes a mental calculation task differs depending on the condition, and as a result, the proportions of each phase in the time length of one trial run also varied depending on the task condition. Due to the high temporal resolution of EEG, the differences between the conditions may have been reflected strongly and may have caused unexpected fluctuations in the data.

By addressing these issues in future research, it would be possible to clarify the indicators of EEG activities that are specific to the Flow condition. For example, it will be possible to identify the transition between the phases during mental calculation tasks and accurately measure the EEG activity of each phase. It will also be effective to use tasks that do not have qualitatively different phase transitions during trials. In addition to the mental calculation tasks used in this research, several experimental paradigms for evoking flow in experimental environment have been proposed. Among these paradigms, tasks that use a gaming framework seem to be promising because they do not necessarily call for a change in the task structure during a single trial. However, in the case of using video games as a task, there is a risk that bodily movement is produced when playing a game affects EEG data; therefore, it is necessary to develop tasks with a minimal amount of bodily movement. Furthermore, in previous studies using video games, task difficulty was not automatically and continuously changed based on the performance of a participant. It is, therefore, necessary to develop a method that enables such adjustments.

In terms of physiological measurements, this research used only EEG. However, the other physiological indicators are considered to be effective tools for establishing objective indicators of the state of flow. de Manzano et al. (2010) reported a positive correlation between the activity of zygomatic major muscle and flow. They regarded this muscle activity as a reflection of positive emotional experiences accompanying the state of flow. On the other hand, although not shown to have any relationship with flow in their results, negative emotional experiences of non-flow state were expected to be reflected by the corrugator supercilii muscle. If negative or positive emotional experiences during the task are actually reflected in these muscular activities, these may provide effective indicators to distinguish Flow and Overload conditions, which have not been clearly distinguished using EEG measurement in this research.

## Conclusion

This research attempted to clarify the EEG activity corresponding to the Flow condition using mental calculation tasks, in which task levels were dynamically adjusted to evoke flow. In conclusion, theta activities in frontal areas were identified to be related to a high level of cognitive control and the immersion aspect of flow. Furthermore, in the flow state, we observed suppressed alpha activity in the frontal and central areas, suggesting that the load on the working memory was not excessive. The revealed EEG activity suggests that a combination of these activities may distinguish the state of flow from other states such as boredom and overload. However, the results of this study are insufficient to establish objective indicators to quantify the strength of flow. Therefore, future research is needed to examine more diverse data and identify the features of physiological activity in the flow state beyond the types of task.

## Author contributions

Conceptualization: KK and NN. Data curation: YY, HO, CY, and SN. Formal analysis: KK, YY, HO, CY, and SN. Funding acquisition: KK and NN. Investigation: YY, HO, CY, and SN. Methodology: KK, HO, CY, SN, and NN. Project administration: NN. Resources: KK and NN. Software: YY, HO, and CY. Supervision: NN. Visualization: KK, HO, and CY. Writing–original draft: KK. Writing–review and editing: YY and NN.

### Conflict of interest statement

The authors declare that the research was conducted in the absence of any commercial or financial relationships that could be construed as a potential conflict of interest.
